# Constitutive Changes in Circulating Follicular Helper T Cells and Their Subsets in Patients with Graves' Disease

**DOI:** 10.1155/2018/8972572

**Published:** 2018-12-24

**Authors:** Yan Liu, Xinwang Yuan, Xiaofang Li, Dawei Cui, Jue Xie

**Affiliations:** ^1^Department of Blood Transfusion, The First Affiliated Hospital, Zhejiang University School of Medicine, Hangzhou 310003, China; ^2^Department of Laboratory Medicine, The First Affiliated Hospital of Zhejiang Chinese Medical University, Hangzhou 310006, China

## Abstract

**Background:**

Follicular helper T (Tfh) cells are critical for high-affinity antibody generation and B cell maturation and differentiation, which play important roles in autoimmune diseases. Graves' disease (GD) is one prototype of common organ-specific autoimmune thyroid diseases (AITD) characterized by autoreactive antibodies, suggesting a possible role for Tfh cells in the pathogenesis of GD. Our objective was to explore the role of circulating Tfh cell subsets and associated plasma cells (PCs) in patients with GD.

**Methods:**

Thirty-six patients with GD and 20 healthy controls (HC) were enrolled in this study. The frequencies of circulating Tfh cell subsets and PCs were determined by flow cytometry, and plasma cytokines, including interleukin- (IL-) 21, IL-4, IL-17A, and interferon- (IFN-) *γ*, were measured using an enzyme-linked immunosorbent assay (ELISA). The mRNA expression of transcription factors (Bcl-6, T-bet, GATA-3, and ROR*γ*t) in peripheral blood mononuclear cells (PBMCs) was evaluated by real-time quantitative PCR*. Results*. Compared with HC, the frequencies of circulating CD4^+^CXCR5^+^CD45RA^−^Tfh (cTfh) cells with ICOS and PD-1 expression, the Tfh2 subset (CXCR3^−^CCR6^−^Tfh) cells, and PCs (CD19^+^CD27^high^CD38^high^) were significantly increased in the GD patients, but the frequencies of Tfh1 (CXCR3^+^CCR6^−^Tfh) and Tfh17 (CXCR3^−^CCR6^+^Tfh) subset cells among CD4^+^T cells were significantly decreased in GD patients. The plasma concentrations of IL-21, IL-4, and IL-17A were elevated in GD patients. Additionally, a positive correlation was found between the frequency of PD-1^+^Tfh cells (Tfh2 or PCs) and plasma IL-21 concentration (or serum TPO-Ab levels). The mRNA levels of transcription factors (GATA-3 and ROR*γ*t) were significantly increased, but T-bet and Bcl-6 mRNA expression was not obviously varied in PBMCs from GD patients. Interestingly, Tfh cell subsets and PCs from GD patients were partly normalized by treatment.

**Conclusion:**

Circulating Tfh cell subsets and PCs might play an important role in the pathogenesis of GD, which are potential clues for GD patients' interventions.

## 1. Introduction

Graves' disease (GD) is a typical organ-specific autoimmune disease that affects the thyroid gland with hyperthyroidism and diffuse goiter [1–3]. It is well known that multiple factors such as environmental triggers, genetic susceptibility, and autoimmune responses are involved in GD [1, 2]. GD, one of autoimmune thyroid diseases (AITD), is characterized by autoantibodies targeted to thyroid tissues, including thyroglobulin antibodies (Tg-Ab), thyroid peroxidase antibodies (TPO-Ab), and thyroid-stimulating hormone receptor antibodies (TR-Ab), as well as by lymphocytic infiltration (T cells and B cells) in thyroid tissue [4, 5]. In thyroid tissues, the interactions among antigen-presenting cells (APCs) and autoactivated T cells and B cells contribute to the production of inflammatory cytokines and autoantibodies that are involved in thyroid gland damage and thyroid dysfunction, which play critical roles in the pathogenesis of GD [2, 5, 6].

Numerous studies have suggested that different CD4^+^T helper (Th) cells, such as Th1, Th2, Th17, and regulatory T (Treg) cells, play important roles in the immunopathogenesis of GD [5, 7–10]. Recently, follicular helper T (Tfh) cells, an important CD4^+^T cell subset associated with the development of germinal centers (GC), B cell differentiation, and antibody production, have also been explored in many autoimmune diseases such as GD, systemic lupus erythematosus (SLE), rheumatoid arthritis (RA), Sjögren's syndrome (SS), and immune thrombocytopenia (ITP) [11–15]. Tfh cells are characterized by the surface expression of CXC chemokine receptor 5 (CXCR5), inducible costimulator (ICOS), and programmed death-1 (PD-1); the expression of the transcription factor B cell lymphoma-6 (Bcl-6); and high-level IL-21 secretion, among other phenotypes [12, 14, 16–18]. More recently, three phenotypic and functionally distinct subsets of blood Tfh cells have been reported based on the differential expression of chemokine receptor 6 (CCR6) and CXC chemokine receptor 3 (CXCR3): CXCR3^+^CCR6^−^Tfh1, CXCR3^−^CCR6^−^Tfh2, and CXCR3^−^CCR6^+^Tfh17 [19–21]. The imbalance in these Tfh cells and subsets plays an important role in the pathogenesis of autoimmune diseases; however, the investigation of these effector Tfh cells and subsets remains not exclusive in GD.

Therefore, we investigated the frequencies of effector-circulating Tfh (cTfh) and cTfh cell subsets (Tfh1, Tfh2, and Tfh17), as well as of plasma cells (PCs), in peripheral blood from patients with GD in this study. We found increased frequencies of effector cTfh cells and Tfh2 subset, as well as PCs in GD patients. Moreover, a positive correlation was observed between circulating Tfh2 (or PD-1^+^Tfh, PCs) cell and serum TPO-Ab levels in GD patients. These findings indicated that cTfh cells and their subsets play crucial roles in the pathogenesis of GD.

## 2. Materials and Methods

### 2.1. Patient Demographics

Based on common clinical and laboratory criteria detailed in previous reports [1], thirty-six newly diagnosed GD patients, including 9 males and 27 females, were enrolled in this study. Patients with GD were treated with methimazole (20–30 mg/day) for the first phase, and the dose was reduced by 5–10 mg/d to reach the maintenance dose when the patients achieved remission. All of the patients received more than 6 months of therapy. The main clinical and laboratory data are presented in Table 1. The serum levels of triiodothyronine (T3), thyroxine (T4), thyroid-stimulating hormone (TSH), free T3 (FT3), free T4 (FT4), Tg-Ab, and TPO-Ab were measured with a Siemens ADVIA Centaur® XP system (Siemens Healthcare Diagnostics, Tarrytown, NY, USA). Twenty age- and sex-matched healthy volunteers were recruited as healthy controls (HC) who did not have a history of autoimmune diseases. The levels of serum T3, T4, FT3, FT4, TSH, Tg-Ab, and TPO-Ab were detected in GD patients and HC. None of the patients had other autoimmune diseases, and they had not received any drugs associated with immunologic diseases. Peripheral blood specimens were obtained from the patients and HC, and their clinical and laboratory data are shown in Table 1.

Written informed consent was obtained from all subjects according to the Declaration of Helsinki (1964), and the study was approved by the local Medical Ethics Committee of the First Affiliated Hospital, Zhejiang University School of Medicine, Zhejiang, China.

### 2.2. Flow Cytometric Analysis

Human fresh peripheral blood specimens were obtained from the GD patients, including 36 patients before treatment and 21 patients after treatment, and HC. The peripheral blood mononuclear cells (PBMCs) were separated immediately by density gradient separation with Ficoll-Hypaque solution (CL5020, CEDARLANE, Canada). Human PBMCs were washed with phosphate-buffered saline (PBS) and immunostained with various antibodies [19]. The following antibodies were used: V450-mouse-anti-human CD4, FITC-mouse-anti-human CD45RA, PerCP-Cy^TM^5.5-rat-anti-human CD185 (CXCR5), PE-mouse-anti-human CD278 (ICOS), APC-mouse-anti-human CD279 (PD-1), APC-mouse-anti-human CD196 (CCR6), PE-mouse-anti-human CD183 (CXCR3), V450-mouse-anti-human CD19, FITC-mouse-anti-human CD138, and APC-mouse-anti-human IgD, as well as isotype-matched antibodies (BD Biosciences, San Diego, CA, USA). Isotype-matched antibody controls were used in all procedures. All the staining procedures were performed according to the manufacturers' protocols. The stained cells were washed with PBS and analyzed by multiparameter flow cytometry (BD FACSVerse™) (Becton Dickinson, Sparks, MD, USA); data were analyzed with FlowJo software, version 7.6.5 (Tree Star Inc., San Carlos, CA, USA). Effector-circulating Tfh cells were defined as CD4^+^CXCR5^+^CD45RA^−^, and three Tfh subsets were also defined based on the expression of CXCR3 and CCR6: CXCR3^+^CCR6^−^Tfh1 cells, CXCR3^−^CCR6^−^Tfh2 cells, and CXCR3^−^CCR6^+^Tfh17 cells. PCs were defined as CD19^+^CD27^high^CD38^high^.

### 2.3. Analysis of Plasma Cytokines

The plasma was separated from all subject samples, and cytokines (IL-21, IL-4, IL-17A, and IFN-*γ*) were measured using an enzyme-linked immunosorbent assay (ELISA) (BioLegend ELISA kit with precoated plates; BioLegend, San Diego, CA, USA) following the manufacturer's protocols.

### 2.4. Real-Time PCR

To detect the mRNA expression levels of the transcription factors Bcl-6, T-bet, GATA-binding protein 3 (GATA-3), and retinoid-related orphan receptor *γ*t (ROR*γ*t), total RNA from PBMCs was extracted using a QIAGEN RNeasy Mini Kit (74104) (QIAGEN GmbH, Hilden, Germany). Subsequently, cDNA was synthesized using a PrimeScript™ II 1st Strand cDNA Synthesis Kit (Takara, Dalian, China) according to the manufacturer's protocol. Real-time PCR was performed utilizing the QuantiFast™ SYBR Green PCR Kit (QIAGEN GmbH, Hilden, Germany) based on the ABI 7500 analysis system (Applied Biosystems, Foster City, CA, USA). The amplification conditions were as follows: 5 min at 95°C for denaturation and 40 cycles (95°C for 10 s and 60°C for 40 s) for PCR amplification. The fluorescence values were collected at 60°C. The primer sequences were as follows: Bcl-6: forward, 5′-CATGCAGAGATGTGCCTCCACA-3′, reverse, 5′-TCAGAGAAGCGGCAGTCACACT-3′ [15]; T-bet: forward, 5′-CGGGAGAACTTTGAGTCC-3′, reverse, 5′-ACTGGTTGGGTAGGAGAGGAG-3′ [22]; GATA-3: forward, 5′-AGACCACCACAACCACACT-3′, reverse, 5′-GATGCCTTCCTTCTTCATAGTCA-3′ [22]; and ROR*γ*t: forward, 5′-GAGGAAGTGACTGGCTACCAGA-3′, reverse, 5′-GCACAATCTGGTCATTCTGGCAG-3′ [23]. Each gene was corrected for variation in samples by the expression of the housekeeping gene glyceraldehyde 3-phosphate dehydrogenase (GAPDH) with the following primers described in a previous report [15]: forward, 5′-GTCTCCTCTGACTTCAACAGCG-3′; reverse, 5′-ACCACCCTGTTGCTGTAGCCAA-3′. The data were analyzed with ABI 7500 software, v2.0.5 (Applied Biosystems, Foster City, CA, USA). The experiments were performed in triplicate in this study.

### 2.5. Statistical Analysis

All data were analyzed with GraphPad Prism 6 software (GraphPad Software, San Diego, CA, USA). All continuous variables are expressed as median ± SD. One-way ANOVA analysis was used to determine the overall statistical significance of multiple-group comparisons. The Mann–Whitney *U*-test was performed for statistical comparisons between the two groups. Correlations of two variables were analyzed by Spearman's correlation coefficient. *p* < 0.05 indicated statistical significance.

## 3. Results

### 3.1. Expanded Frequency of Circulating Tfh Cells in Patients with GD

**Figure 1:**
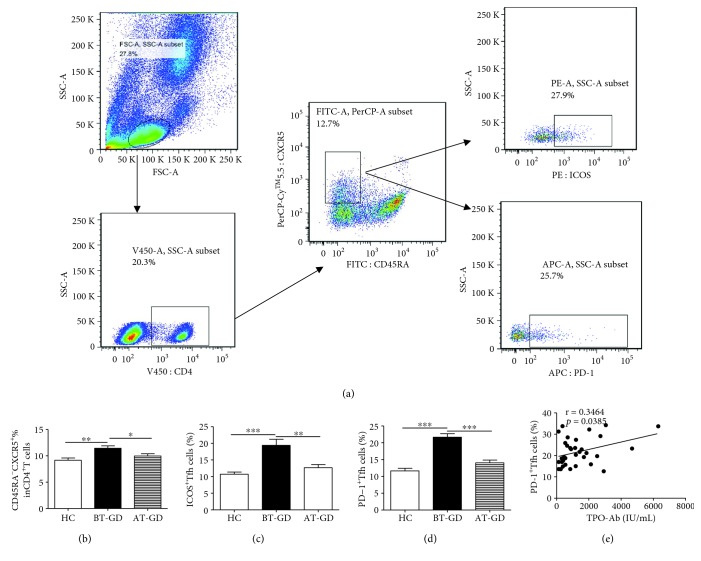
Flow analysis of circulating Tfh cells in GD patients. Human PBMCs from GD patients (BT: 36; AT: 21) and 20 HC were stained with anti-CD4, anti-CXCR5, anti-ICOS, anti-CD45RA, and anti-PD-1. (a) The cells were gated initially on lymphocytes and then circulating Tfh cells were analyzed by flow cytometry; (b) the numbers of circulating CD4^+^CXCR5^+^CD45RA^−^Tfh (cTfh) cells; (c) the numbers of CD4^+^CXCR5^+^CD45RA^−^ICOS^+^T cells; (d) CD4^+^CXCR5^+^CD45RA^−^PD-1^+^Tfh cells; (e) the correlation between PD-1^+^Tfh cell proportions and TPO-Ab levels in GD patients. ^∗^*p* < 0.05, ^∗∗^*p* < 0.01, and ^∗∗∗^*p* < 0.001; ns: no significant difference.

To investigate the potential role of effector cTfh cells in peripheral blood from patients with GD, the frequencies of circulating CD4^+^CXCR5^+^CD45RA^−^Tfh (cTfh) cells were analyzed by flow cytometry (Figure 1(a)). The frequencies of cTfh cells were significantly increased in patients in the GD before treatment (BT-GD) group compared to those in HC (Figure 1(b)). Moreover, the frequencies of PD-1^+^Tfh cells and ICOS^+^Tfh cells were notably expanded in patients with GD (Figures 1(c) and 1(d)). Interestingly, PD-1^+^Tfh cells (not ICOS^+^Tfh cells) were closely correlated with high serum levels of TPO-Ab from the GD patients (Figure 1(e)). Additionally, there was no correlation between the PD-1^+^Tfh and ICOS^+^Tfh cells in patients with GD (data not shown). The frequency of cTfh cells from some GD patients partly normalized after treatment (AT-GD), and there were no differences between AT-GD and HC groups (Figures 1(b)–1(d)).

### 3.2. Increased Tfh2 Cells Are the Predominant Tfh Cell Subsets in GD Patients

**Figure 2:**
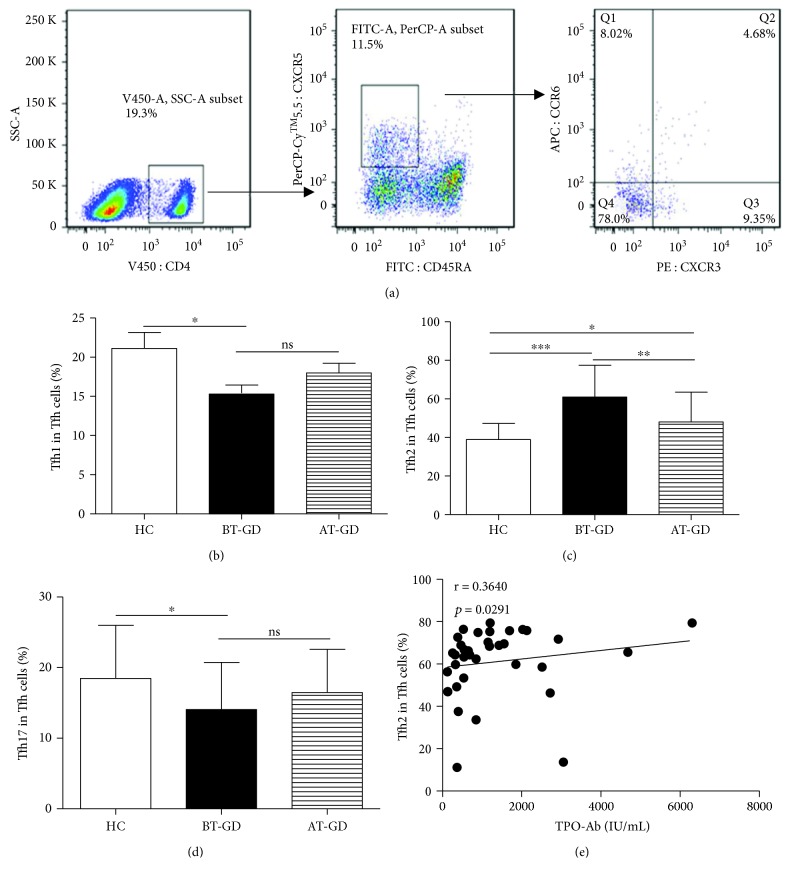
Frequency of circulating Tfh cell subsets in GD patients. (a) Representative dot plots demonstrate CXCR3 and CCR6 expression in cells gated for CD4, CD45RA, and CXCR5; (b) lower proportions of Tfh1 cells in GD patients; (c) overabundance of Tfh2 cells in GD patients; (d) decreased Tfh17 cells in GD patients; (e) relation of Tfh2 subset proportions with levels of serum TPO-Ab in GD patients. ^∗^*p* < 0.05, ^∗∗^*p* < 0.01, and ^∗∗∗^*p* < 0.001; ns: no significant difference. Tfh1 cells, CXCR3^+^CCR6^−^Tfh cells; Tfh17 cells, CXCR3^−^CCR6^+^Tfh cells; Tfh2 cells, CXCR3^−^CCR6^−^Tfh cells. GD patients (BT: 36, AT: 21) and 20 HC were enrolled in this study.

Blood Tfh cells can be further classified into three distinct subsets depending on chemokine receptors on the cell surface: Tfh1 (CXCR3^+^CCR6^−^), Tfh2 (CXCR3^−^CCR6^−^), and Tfh17 (CXCR3^−^CCR6^+^) (Figure 2(a)). Among the cTfh cells, Tfh2 cells were the majorly increased subset; the frequencies of Tfh17 and Tfh1 cells were significantly decreased in GD patients compared with HC, although there were no differences about Tfh1 or Tfh17 cell frequencies between the BT-GD and AT-GD groups (Figures 2(b)–2(d)). Additionally, the proportion of Tfh2 cells was positively correlated with high levels of TPO-Ab in GD patients without treatment (Figure 2(e)). The frequency of cTfh cell subsets from some GD patients partly normalized after treatment, and there were no differences about Tfh1 or Tfh17 cell frequencies between the AT-GD and HC groups (Figures 2(b)–2(d)).

### 3.3. Frequency of Circulating Plasma Cells Expanded in GD Patients

**Figure 3:**
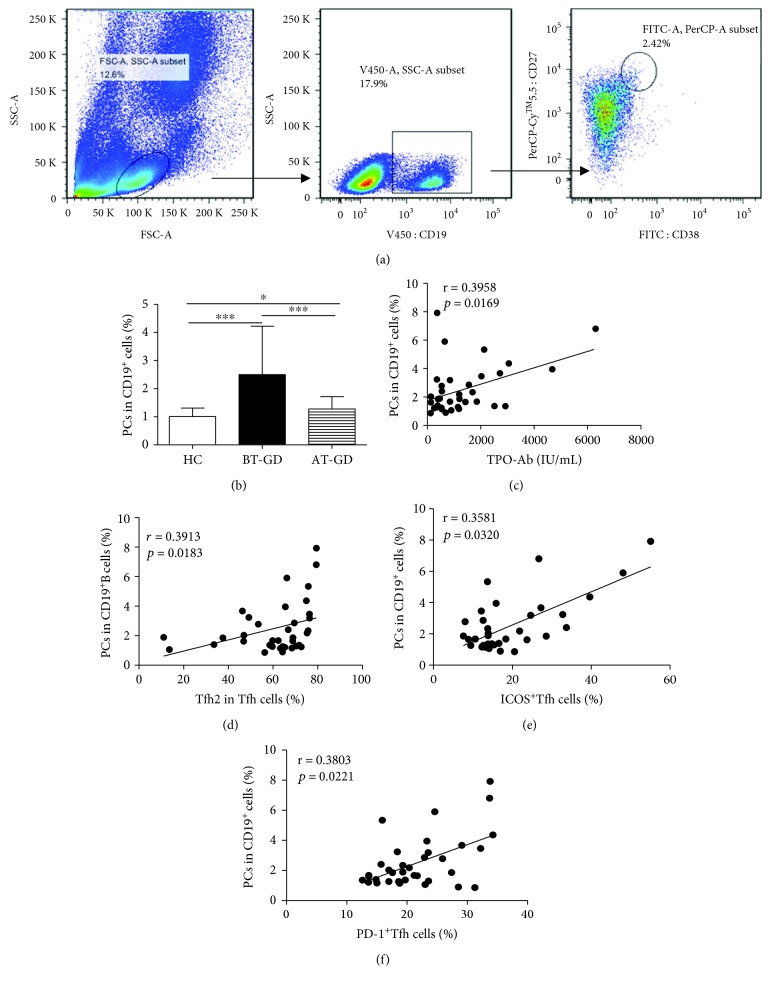
Expanded frequency of circulating plasma cells in GD patients. Human PBMCs from GD patients (BT: 36; AT: 21) and 20 HC were stained with anti-CD19, anti-CD38, and anti-CD27. (a) The cells were gated initially on lymphocytes and then on CD19^+^B cells; afterwards, the numbers of CD27^high^CD38^high^ PCs were analyzed by flow cytometry. (b) Changes in PCs in GD patients. (c) Relation of PC proportions and serum TPO-Ab levels in GD patients. (d) Relation of Tfh2 subset proportions with PCs in GD patients. (e) The correlation between the proportions of PCs and ICOS^+^Tfh cells in GD patients. (f) The correlation between the proportions of PCs and PD-1^+^Tfh cells in GD patients. ^∗^*p* < 0.05, ^∗∗^*p* < 0.01, and ^∗∗∗^*p* < 0.001; ns: no significant difference.

The number of circulating PCs (CD19^+^CD27^high^CD38^high^) was analyzed by flow cytometry (Figure 3(a)). The frequencies of circulating PCs were significantly increased in patients with GD compared with HC (Figure 3(b)). Interestingly, the frequency of circulating PCs was positively correlated not only with the frequency of serum TPO-Ab level but also with Tfh2 cells in GD patients (Figures 3(c) and 3(d)). In addition, there was a positive correlation between the proportions of circulating PCs and frequencies of ICOS^+^Tfh (or PD-1^+^Tfh) in GD patients (Figures 3(e) and 3(f)). The frequency of PCs from some GD patients was significantly decreased after treatment compared to the BT-GD group, but higher than that from the HC group (Figure 3(b)).

### 3.4. Plasma Cytokine Secretion Profile in GD Patients

**Figure 4:**
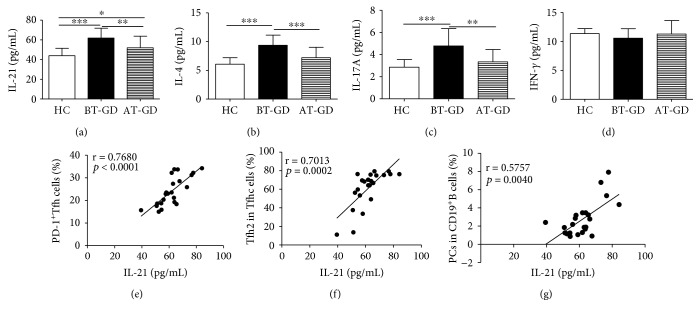
Plasma cytokine levels in GD patients. (a–d) The levels of plasma IL-21, IL-4, IL-17A, and IFN-*γ* were detected by ELISA in GD patients (BT: 23; AT: 16) and 20 HC. (e) Relation of PD-1^+^Tfh with IL-21. (f) Relation of Tfh2 frequency with IL-21 level. (g) Relation of PC frequency with IL-21 level. ^∗^*p* < 0.05, ^∗∗^*p* < 0.01, and ^∗∗∗^*p* < 0.001; ns: no significant difference.

To explore the potential value of Tfh cell subsets associated with plasma cytokines in GD patients, plasma concentrations of several cytokines (IL-21, IL-4, IL-17A, and IFN-*γ*) from the patients and HC were measured with an ELISA assay. The plasma concentrations of IL-21, IL-4, and IL-17A were significantly elevated in BT-GD patients, the levels of these cytokines from some GD patients were significantly reduced in the AT-GD group compared to the BT-GD group, and there was no significant difference about the IL-4 and IL-17A cytokines between the AT-GD and HC groups except the plasma IL-21 level (Figures 4(a)–4(c)). However, the plasma IFN-*γ* concentration was not notably changed in GD patients compared to HC (Figure 4(d)). Additionally, a positive correlation was observed between the levels of IL-21 and the frequencies of circulating PD-1^+^Tfh (or Tfh2 cells, PCs) in GD patients (Figures 4(e)–4(g)).

### 3.5. Expression Profiles of mRNA in Peripheral PBMCs from GD Patients

**Figure 5:**
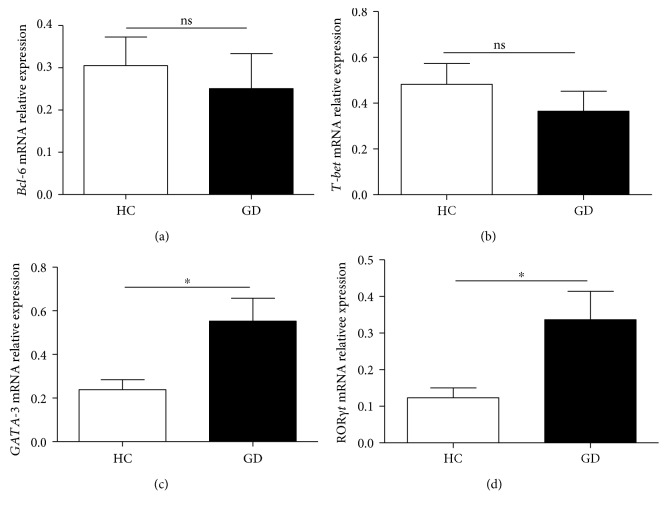
mRNA expression in human CD4^+^T cells from GD patients. The expression of several transcription factors (Bcl-6, T-bet, GATA-3, and ROR*γ*t) in peripheral CD4^+^T cells from the 9 GD patients and 12 HC was detected with a real-time PCR assay. (a) Relative expression levels of Bcl-6 mRNA; (b) relative expression levels of T-bet mRNA; (c) relative expression levels of GATA-3 mRNA; (d) relative expression levels of ROR*γ*t mRNA. ^∗^*p* < 0.05, ^∗∗^*p* < 0.01, and ^∗∗∗^*p* < 0.001; ns: no significant difference.

Next, to further elucidate the role of cTfh cells and subsets in GD patients, the mRNA expression levels of several transcription factors (Bcl-6, T-bet, GATA-3, and ROR*γ*t) in PBMCs from the nine patients and eight HC were detected with a real-time PCR assay. The results indicated that the levels of Bcl-6, GATA-3, and ROR*γ*t mRNA were significantly increased in GD patients compared with those in HC (Figures 5(a)–5(c)). However, in comparison with that in HC, the level of T-bet mRNA was not obviously changed in GD patients (Figure 5(d)).

## 4. Discussion

GD is one of the typical organ-specific autoimmune diseases which is characterized by thyroid autoantibodies and autoreactive lymphocytes in thyroid tissues [1, 2, 24]. It is well known that the cellular and humoral immune mechanisms are closely correlated with the pathogenesis of autoimmune disorders such as RA and SLE [5, 25, 26]. Recent evidence has indicated that the complex mechanisms of the interactions among APCs, especially DCs, T cells, B cells, and autoantibodies, are involved in GD, although autoantibodies are very critical for the pathogenesis of GD [2–5, 10]. In this study, the levels of serum Tg-Ab and TPO-Ab in GD patients were significantly elevated, and the concentration of TSH in GD patients was notably decreased compared to that in HC. These results indicated that antibodies were associated with the pathogenesis of GD, which corresponded with previous reports [9, 27].

Recently, Tfh cells that were differentially defined as CD4^+^CXCR5^+^ICOS^high^ or PD-1^high^Tfh cells or CD4^+^CXCR5^+^CD45RA^−^ or CD4^+^CXCR5^+^CD45RA^−^PD-1^+^Tfh cells were all significantly expanded and contributed to the production of autoantibodies in autoimmune diseases such as AITD, RA, SLE, and IgG4-related diseases (IgG4-RD) [20, 21, 27–30]. Moreover, the levels of CD4^+^CXCR5^+^Tfh cells were significantly elevated in peripheral blood or thyroid tissues from GD patients compared with healthy controls [31, 32]. In this study, the percentages of circulating CD4^+^CXCR5^+^CD45RA^−^Tfh (cTfh), PD-1^+^Tfh, and ICOS^+^Tfh cells in the peripheral blood were significantly increased in GD patients compared to those in HC; these results were consistent with previous reports [21, 27, 31, 32]. Additionally, the proportion of PD-1^+^Tfh cells (not ICOS^+^Tfh cells) had a positive relation with serum TPO-Ab level (or PC frequency) in GD patients, and no relation between PD-1^+^Tfh and ICOS^+^Tfh cells was observed in patients with GD, which was partly different from a previous report [21]. These discrepancies might be due to the definition of cTfh cells that affected the data analysis in our study. Together, these findings indicated that cTfh, PD-1^+^Tfh, and ICOS^+^Tfh cells likely contribute to autoantibody production and are involved in the pathogenesis of GD.

Three distinct subsets of Tfh cells were defined depending on the expression of CXCR3 and CCR6: CXCR3^+^CCR6^−^Tfh1 cells, CXCR3^−^CCR6^−^Tfh2 cells, and CXCR3^−^CCR6^+^Tfh17 cells [20, 21]. Tfh1 cells share similar characteristics with Th1 cells that express the transcription factor T-bet, and they secrete the cytokine IFN-*γ*; Tfh2 cells express the transcription factor GATA-3 and exclusively produce the Th2 cytokines IL-4, IL-5, and IL-13; and Tfh17 cells share similar characteristics with Th17 cells that express the transcription factor ROR*γ*t and produce the cytokines IL-17A and IL-22 [21, 33, 34]. Recent evidence has shown that human Tfh2 and Tfh17 cells, not Tfh1 cells, can efficiently help B cells produce immunoglobulins (Igs) and differentially regulate isotype switches *in vitro* [20, 21]. Accumulating evidence has indicated that alterations of Tfh1, Tfh2, and Tfh17 cells are closely related with the pathogenesis of autoimmune diseases, such as myasthenia gravis, IgG4-RD, and SLE [35–37]. Our study showed a decreased frequency of Tfh1 and Tfh2 cells and increased proportions of Tfh2 cells in peripheral blood from GD patients, and Tfh2 cells were the majority of the three distinct Tfh cell subsets. Furthermore, the alteration of Tfh2 cells was closely correlated with serum TPO-Ab titers and the increased frequency of blood PCs in GD patients. These findings indicated that the imbalance of Tfh cell subsets was involved in the pathogenesis of GD, and Tfh2 cells were more efficient in inducing B cell differentiation into PCs that produce high antibodies in GD patients.

Accumulating evidence has shown that Tfh cells are characterized by the transcription factor Bcl-6 and abundant IL-21 secretion, and the cytokine IL-21 is involved in the proliferation and differentiation of Tfh cells and PCs and in antibody responses [33, 38–41]. In our study, the mRNA expression level of the transcription factor Bcl-6 in blood PBMC was not notably varied, but the plasma levels of IL-21 were significantly increased in GD patients compared with those in HC. Moreover, the plasma concentration of IL-21 had a positive correlation with the frequency of blood PD-1^+^Tfh cells (or PCs, Tfh2 cell) in GD patients. These findings implied that IL-21 contributed to PD-1^+^Tfh, Tfh2 cell, and PC generation and differentiation. Transcription factors were possibly associated with memory Tfh cell in GD patients in our study; these findings were consistent with previous studies [27, 31, 32, 42].

Additionally, three distinct Tfh cell subset-associated cytokines/transcription factors were analyzed in our study. Previous studies have demonstrated the important roles of Th1, Th2, and Th17 cells and their associated cytokines/transcription factors in GD patients [1, 2, 5, 8–10, 43]. Specific IL-4-producing Tfh2 cells are important cytokines for Ig production and class switching [21]. In this study, no correlation between increased plasma IL-4 levels and the percentages of blood Tfh2 cells implied that IL-4 cytokine was not only from Tfh2 cells but also from other immune cells as Th2 cells in GD patients. The mRNA expression levels of the transcription factor GATA-3 in blood PBMCs were significantly increased in GD patients compared with HC, which implied that GATA-3 might be associated with Tfh2 cell generation and differentiation in GD patients. IL-17A originates from various immune cells, such as Th17, Tfh17, and CD8^+^T cells, which contribute to tissue damage in GD and other autoimmune diseases [9, 10, 21]. Plasma levels of IL-17A, as well as the mRNA expression of ROR*γ*t in PBMCs, were also increased in GD patients compared with HC. The IFN-*γ* cytokine is produced by Th1, Tfh1, CD8^+^T, *γδ*T, NK cells, and other immune cells and is associated with subsequent thyroiditis and thyroid gland destruction [5, 8, 21]. However, plasma levels of cytokine IFN-*γ* and the mRNA expression of T-bet in PBMCs were not notably altered in GD patients compared with those in HC. The plasma levels of cytokines (IL-21, IL-4, IL-17A, and IFN-*γ*) and the mRNA expression of transcription factors (Bcl-6, T-bet, GATA-3, and ROR*γ*t) in blood PBMCs from GD patients were significantly different from those of HC, which implied that these cytokines and transcription factors played important roles in the pathogenesis of GD. Taken together, these findings indicated that the distinct subsets of blood cTfh cells and associated cytokines/transcription factors were involved in the generation of PCs that secrete autoantibodies and participated in the pathogenesis of GD, which will provide valuable insights into potential therapeutic strategies for human autoimmune diseases.

## 5. Conclusion

In summary, we show that circulating PD-1^+^Tfh and ICOS^+^Tfh cells are expanded together with Tfh2 cell subsets, associated cytokines, and transcription factors in peripheral blood from GD patients. Excessive blood cTfh cells and Tfh subsets can contribute to PC generation and antibody production in GD patients. These findings indicate that Tfh cells and subsets play an important role in the pathogenesis of GD and that they are potential valuable therapeutic targets for human GD. However, the precise role of these Tfh cells and subsets, as well as their interactions with B cells and other immune cells, remains to be further explored in the pathogenesis GD.

## Figures and Tables

**Table 1:** Main characteristics of patients with GD included in this study.

	BT	AT	Range
Number	36	21	
Gender (M/F)	9/27	4/17	
Age (years)	36.33 ± 14.08	36.83 ± 14.08	
T3 (nmol/L)	3.36 ± 2.70	2.15 ± 0.78	1.02-2.96
T4 (nmol/L)	155.43 ± 91.10	113.99 ± 26.25	55.47-161.25
FT3 (pmol/L)	11.13 ± 8.70	4.24 ± 0.47	2.77-6.31
FT4 (pmol/L)	29.01 ± 25.56	15.01 ± 5.23	10.45-24.38
TSH (mIU/L)	0.032 ± 0.040	1.809 ± 1.534	0.380-4.340
Tg-Ab (U/mL)	207.49 ± 242.63	65.46 ± 48.52	<60
TPO-Ab (IU/mL)	1225.67 ± 1275.55	711.59 ± 1237.49	<100

Note: data are presented as mean ± standard deviation. M/F: male/female; BT: before treatment with new diagnosis; AT: after treatment; TSH: thyroid-stimulating hormone; T3: triiodothyronine; T4: thyroxine; FT3: free T3; FT4: free T4; Tg-Ab: thyroglobulin antibody; TPO-Ab: thyroperoxidase antibody.

## Data Availability

The data used to support the findings of this study are included within the article.

## References

[1] Antonelli A., Ferrari S. M., Corrado A., di Domenicantonio A., Fallahi P. (2015). Autoimmune thyroid disorders. *Autoimmunity Reviews*.

[2] Yoo W. S., Chung H. K. (2016). Recent advances in autoimmune thyroid diseases. *Endocrinology and Metabolism*.

[3] Lee H. J., Li C. W., Hammerstad S. S., Stefan M., Tomer Y. (2015). Immunogenetics of autoimmune thyroid diseases: a comprehensive review. *Journal of Autoimmunity*.

[4] Fröhlich E., Wahl R. (2017). Thyroid autoimmunity: role of anti-thyroid antibodies in thyroid and extra-thyroidal diseases. *Frontiers in Immunology*.

[5] Ramos-Leví A. M., Marazuela M. (2016). Pathogenesis of thyroid autoimmune disease: the role of cellular mechanisms. *Endocrinología y Nutrición*.

[6] Wang B., Shao X., Song R., Xu D., Zhang J.-a. (2017). The emerging role of epigenetics in autoimmune thyroid diseases. *Frontiers in Immunology*.

[7] Figueroa-Vega N., Alfonso-Pérez M., Benedicto I., Sánchez-Madrid F., González-Amaro R., Marazuela M. (2010). Increased circulating pro-inflammatory cytokines and Th17 lymphocytes in Hashimoto’s thyroiditis. *The Journal of Clinical Endocrinology and Metabolism*.

[8] Inoue N., Watanabe M., Nakaguchi A. (2017). Functional polymorphisms affecting Th1 differentiation are associated with the severity of autoimmune thyroid diseases. *Endocrine Journal*.

[9] Li C., Yuan J., Zhu Y. F. (2016). Imbalance of Th17/Treg in different subtypes of autoimmune thyroid diseases. *Cellular Physiology and Biochemistry*.

[10] Gonzalez-Amaro R., Marazuela M. (2016). T regulatory (Treg) and T helper 17 (Th17) lymphocytes in thyroid autoimmunity. *Endocrine*.

[11] Nurieva R. I., Chung Y., Hwang D. (2008). Generation of T follicular helper cells is mediated by interleukin-21 but independent of T helper 1, 2, or 17 cell lineages. *Immunity*.

[12] Fazilleau N., Mark L., McHeyzer-Williams L. J., McHeyzer-Williams M. G. (2009). Follicular helper T cells: lineage and location. *Immunity*.

[13] Gómez-Martin D., Díaz-Zamudio M., Romo-Tena J., Ibarra-Sánchez M. J., Alcocer-Varela J. (2011). Follicular helper T cells poise immune responses to the development of autoimmune pathology. *Autoimmunity Reviews*.

[14] Vinuesa C. G., Linterman M. A., Yu D., MacLennan I. C. M. (2016). Follicular helper T cells. *Annual Review of Immunology*.

[15] Xie J., Cui D., Liu Y. (2015). Changes in follicular helper T cells in idiopathic thrombocytopenic purpura patients. *International Journal of Biological Sciences*.

[16] Nurieva R. I., Chung Y., Martinez G. J. (2009). Bcl 6 mediates the development of T follicular helper cells. *Science*.

[17] Crotty S. (2011). Follicular helper CD4 T cells (TFH). *Annual Review of Immunology*.

[18] Yu D., Vinuesa C. G. (2010). The elusive identity of T follicular helper cells. *Trends in Immunology*.

[19] Tian X., Ma J., Wang T. (2018). Long non-coding RNA HOXA transcript antisense RNA myeloid-specific 1-HOXA1 axis downregulates the immunosuppressive activity of myeloid-derived suppressor cells in lung cancer. *Frontiers in Immunology*.

[20] Morita R., Schmitt N., Bentebibel S. E. (2011). Human blood CXCR5(+)CD4(+) T cells are counterparts of T follicular cells and contain specific subsets that differentially support antibody secretion. *Immunity*.

[21] Schmitt N., Bentebibel S. E., Ueno H. (2014). Phenotype and functions of memory Tfh cells in human blood. *Trends in Immunology*.

[22] Gao J., Wu Y., Su Z. (2014). Infiltration of alternatively activated macrophages in cancer tissue is associated with MDSC and Th2 polarization in patients with esophageal cancer. *PLoS One*.

[23] Cui D., Zhong F., Lin J. (2017). Changes of circulating Th22 cells in children with hand, foot, and mouth disease caused by enterovirus 71 infection. *Oncotarget*.

[24] Mikoś H., Mikoś M., Obara-Moszyńska M., Niedziela M. (2014). The role of the immune system and cytokines involved in the pathogenesis of autoimmune thyroid disease (AITD). *Endokrynologia Polska*.

[25] Nagy G., Huszthy P. C., Fossum E., Konttinen Y., Nakken B., Szodoray P. (2015). Selected aspects in the pathogenesis of autoimmune diseases. *Mediators of Inflammation*.

[26] Atassi M. Z., Casali P., Atassi M. Z., Casali P. (2008). Molecular mechanisms of autoimmunity. *Autoimmunity*.

[27] Zhu C., Ma J., Liu Y. (2012). Increased frequency of follicular helper T cells in patients with autoimmune thyroid disease. *The Journal of Clinical Endocrinology and Metabolism*.

[28] Akiyama M., Suzuki K., Yamaoka K. (2015). Number of circulating follicular helper 2 T cells correlates with IgG4 and interleukin-4 levels and plasmablast numbers in IgG4-related disease. *Arthritis & Rhematology*.

[29] Arroyo-Villa I., Bautista-Caro M. B., Balsa A. (2014). Constitutively altered frequencies of circulating follicullar helper T cell counterparts and their subsets in rheumatoid arthritis. *Arthritis Research & Therapy*.

[30] Choi J. Y., Ho J. H. E., Pasoto S. G. (2015). Circulating follicular helper-like T cells in systemic lupus erythematosus: association with disease activity. *Arthritis & Rhematology*.

[31] Zhang J., Ren M., Zeng H. (2015). Elevated follicular helper T cells and expression of IL-21 in thyroid tissues are involved in the pathogenesis of Graves’ disease. *Immunologic Research*.

[32] Chen J., Tian J., Tang X. (2015). MiR-346 regulates CD4(+)CXCR5(+) T cells in the pathogenesis of Graves’ disease. *Endocrine*.

[33] Park H. J., Kim D. H., Lim S. H. (2014). Insights into the role of follicular helper T cells in autoimmunity. *Immune Network*.

[34] Che Y., Qiu J., Jin T., Yin F., Li M., Jiang Y. (2016). Circulating memory T follicular helper subsets, Tfh2 and Tfh17, participate in the pathogenesis of Guillain-Barré syndrome. *Scientific Reports*.

[35] Zhang C. J., Gong Y., Zhu W. (2016). Augmentation of circulating follicular helper T cells and their impact on autoreactive B cells in myasthenia gravis. *Journal of Immunology*.

[36] Grados A., Ebbo M., Piperoglou C. (2017). T cell polarization toward T_H_2/T_FH_2 and T_H_17/T_FH_17 in patients with IgG4-related disease. *Frontiers in Immunology*.

[37] Le Coz C., Joublin A., Pasquali J.-L., Korganow A.-S., Dumortier H., Monneaux F. (2013). Circulating T_FH_ subset distribution is strongly affected in lupus patients with an active disease. *PLoS One*.

[38] Xie J., Liu Y., Wang L. (2015). Expansion of circulating T follicular helper cells in children with acute Henoch-Schönlein purpura. *Journal of Immunology Research*.

[39] Crotty S. (2014). T follicular helper cell differentiation, function, and roles in disease. *Immunity*.

[40] Spolski R., Leonard W. J. (2010). IL-21 and T follicular helper cells. *International Immunology*.

[41] Ueno H., Banchereau J., Vinuesa C. G. (2015). Pathophysiology of T follicular helper cells in humans and mice. *Nature Immunology*.

[42] Guan L. J., Wang X., Meng S. (2015). Increased IL-21/IL-21R expression and its proinflammatory effects in autoimmune thyroid disease. *Cytokine*.

[43] Nanba T., Watanabe M., Inoue N., Iwatani Y. (2009). Increases of the Th1/Th2 cell ratio in severe Hashimoto’s disease and in the proportion of Th17 cells in intractable Graves’ disease. *Thyroid*.

